# Is Pollution a Cost to Health? Theoretical and Empirical Inquiry for the World’s Leading Polluting Economies

**DOI:** 10.3390/ijerph18126624

**Published:** 2021-06-20

**Authors:** Ramesh Chandra Das, Enrico Ivaldi

**Affiliations:** 1 Department of Economics, Vidyasagar University, Midnapore 721102, West Bengal, India; ramesh051073@gmail.com; 2 Department of Political Science, University of Genoa, 16125 Genoa, Italy

**Keywords:** pollution, health expenditure, income, EKC, endogenous growth, cointegration, causality

## Abstract

Making development sustainable in the long run is the goal of policy makers of countries all over the world. To attain such a goal, countries have to face the dynamics of pollution-income interactions in both the short and long run, which are observed along the well-known Environmental Kuznets Curve (EKC). In the short run stage of the EKC, rising income and rising health expenditure may lead to rising pollution, while in the long run, as pollution continues, health expenditures increase, besides conservation of capital investment. The former is a common phenomenon in developing economies and the latter in the developed economies. Hence, there are both theoretical and empirical questions on whether health expenditures are caused by environmental pollution or not. The present study has attempted to investigate the issue from the theoretical point of view, through the endogenous growth framework, and by considering empirical observations for the world’s top 20 polluting countries for the period 1991–2019. The results show that per capita health expenditure and per capita pollution are cointegrated in the majority of the countries. However, in the short run, pollution is the cause of health expenditures for many developed countries in the list, and health expenditures are the cause of pollution in some of the developing countries. The results justify the claim of the endogenous growth model incorporating pollution and health expenditure.

## 1. Introduction

Countries’ progress is aimed at the long term goal of sustainable development, which provides all sorts of benefits to all generations of people without worsening the stock of in situ environmental resources on the one hand, and maintaining equitable distribution of their wealth across all classes of people on the other. However, in order to achieve this long-term goal, countries have to take into account the growth and fluctuations of the economic, environmental and social indicators in the short term. Keeping all other factors unchanged, countries can have negative environmental impacts in the short run to achieve sustainable development in the long run. Therefore, dynamic linkages exist in the short and long run among all the socio-economic and environmental variables concerned. Three such important variables are income, environmental pollution and health expenditure, which are the focus of the present study [1,2,3,4,5].

Economies burn fossil fuels for their industrial and tertiary activities to generate more output and income, with negative consequences for the environment in terms of more pollution. This is the short run effect. Furthermore, as income grows in aggregate and per capita terms, people become more conscious about their health conditions and the conservation of the environment. Hence, they buy health care services and invest in environmental protection. Besides, national governments increase spending to provide health care services and conserving nature as part of their policy objectives. These spending on personal health and the environment become part of the long run effect that paves the way for sustainable development.

The question arises, then, of whether health expenditure is a result of environmental degradation or pollution. To answer the question it is necessary to point out the reasons behind health expenditures in an economy. There are two such reasons: one is demand-based and the other is supply-or production-based. As for the demand-based reason, when the economy grows in the early stages, more pollution is generated, people’s income increases and they spend on health care, among other things. On the other hand, as regarding the supply-based reason, production of more goods and services leads to more pollution and in order to protect themselves from the negative consequences of such pollution, people spend money on health care services. The first one is the pull effect and the second one is the push effect. We can link these two effects with the well-known Environmental Kuznets Curve (EKC). EKC is a reversed U-shaped curve in general instances [1,3,6]. The rising part of the EKC can be linked to the demand-based effect or short run effect and the descending part can be linked to the supply-based or long run effect. Therefore, the overall effect of pollution upon health depends on the position of the economy considered in the EKC. It is expected to see that the so-called developed economies are in the descending part of the EKC and the developing economies are in the rising part. In the rising part it is likely to see that income or associated health expenditure may cause the pollution level, and in the descending part it is likely to see that pollution causes health expenditures. It is now both a theoretical and empirical question whether health expenditures are influenced by environmental pollution or not.

## 2. Literature Review

The following is a review of studies in the fields related to our aim. First we present works on health and human capital formation, then on human capital formation and growth and finally on pollution, growth and health expenditure.

Bloom and Canning [7] argue that health, like education, is an essential constituent of human capital, and suggest that better health conditions lead to higher income. According to the World Health Organization (WHO, Geneva, Switzerland) [8], health is not only the absence of illness, but also the capacity of developing a person’s potential throughout his/her life. In this sense, health is an asset or capital with both intrinsic and instrumental values. The roles of micro and macroeconomic factors behind health development and human capital formation have been well identified by Bleakley [9]. His study discussed a range of micro and macro evidence and shows that health is both human capital itself and an input to producing other forms of human capital. The role of government spending on health infrastructure development cannot be ignored in developing human capital. Raghupathi and Raghupathi [10] discovered the degree of interrelationships between government health expenditure and economic performance across the United States on the presumption that healthcare spending leads to better provision of health opportunities. Using visual analytics, the results adequately suggest a positive association between healthcare expenditure and economic indicators such as income, Gross Domestic Product (GDP), and labour productivity.

How does human capital or the educational attainment of the labour force affect the output and the growth of an economy? The study by Mankiw et al. [11] follows a standard practice which is to treat human capital, or the average years of schooling of the labour force, as an ordinary input in the production function. An alternative approach to capture its role in economic growth is to model technological progress as a function of human capital defined by the level of education. Benhabib and Spiegel [12] attempted to distinguish between these two approaches empirically. The study found significant results when including the impact of some ancillary variables-such as absence of political stability and income disparity-on economic growth and factor accrual upon the human capital-growth linkages. How much an economy should spend on health capital formation has not been addressed yet. Chang and Ying [13] have investigated it from theoretical and empirical perspectives in the fifteen OECD (Organisation for Economic Co-operation and Development) countries within the neoclassical growth framework. The theoretical model reveals that there is convergence between the poorer and the wealthier countries when both physical and health capitals are taken into account. However, the empirical analyses show unnecessary health spending in most countries for the past twenty years approximately. Using panel data for 118 developing economies in the 1971–2000 period, Baldacci et al. [14] explore the routes connecting social expenditures, human capital formation, and growth, and compare the impacts of alternative economic policy interventions. They observe that both education and health expenditures have a positive and significant impact on education and health capital formation, and therefore support higher growth. Pelinescu [15] commented that the EU 2020 Strategy—which characterizes growth as smart, sustainable and inclusive—couldn’t be attained without a major influence of assistance, information or value of the people, commonly known as human capital. Using a panel methodology, the study revealed a positive and statistically significant relationship between per capita income and innovative capacity of human capital (measured by patents owned), and qualification of employees.

The EKC hypothesis consisting of the income-pollution relationships has been verified both theoretically and empirically by several studies. We present here a list of them to show the scope of our study.

In this relationship, out of many explanatory variables, income has regularly had the most significant effect on indicators of environmental quality. One variable consistently omitted in these relationships is the price of energy. Agras and Chapman [1] analyse the income-pollution relationships to show the implications of prices in the models. These long-run price-income models show that income is no more the most pertinent determinant of environmental quality. Indeed, they did not observe any significant evidence of the existence of an EKC. Grossman and Krueger [16] link trade restrictions with the emissions of different pollutants and their relative effects upon income in different urban areas in the USA and 42 other countries including Mexico, in order to examine the relationship between air quality and economic growth. They found that the concentrations of two pollutants, sulphur dioxide and “smoke”, increase with per capita GDP at low levels of national income, but decrease with growth of the GDP at larger income levels. Nemat [2] analysed the associations between the level of economic development and quality of the environment for a sizeable sample of countries over time. The results reveal that some indicators, like water and sanitation, improve with increasing incomes, others like particulates and sulphur oxides deteriorate and then progress, and others like dissolved oxygen in river basins, municipal solid wastes, and carbon emissions deteriorate gradually. In their empirical investigation, Dinda et al. [3] detected an inverse relationship between environmental dilapidation and real income per capita, in contrast with the usual inverted U-shaped environmental Kuznets curve found in many earlier studies. A clarification of the observed outline of the relationship might be required in the dynamics of the process of economic growth practised by the concerned countries, and yet studies neglected the issue of pollution having both flow and stock features. Lieb [6], in an overlapping generations model, offers a likely clarification for the empirical result that the pollution-income relationship for the flow pollutants is an EKC, while the pollution-income association for the stock pollutants is monotonically rising. Narayan and Narayan [4] conducted an empirical analysis to find whether environmental quality influences per capita health expenditures. The study takes a panel cointegration approach for eight OECD countries for the period 1980–1999 and it shows that there are co-movements in the panel of per capita health spending, income per capita, carbon monoxide emissions, sulphur oxide emissions and nitrogen oxide emissions. In their work, Baltagi and Moscone [5] revisited the long run economic association between health care expenditure and income using a panel of twenty OECD countries for the 1971–2004 period and suggest that health care and income have long run association and that health care is an inevitability rather than a luxury. In a wider country base, Lago-Penas et al. [17] analysed the interrelationship between health expenditure and income in 31 OECD countries and demonstrated that, in the long-run, health expenditures are more highly sensitive to per capita income’s cyclical movements than to the trend movements. In an extensive study of Narayan and Narayan [4], Chaabouni et al. [18] inspected the causal interplays between CO_2_ emissions, health expenditures and economic growth in a global panel of 51 countries and different income groups for the 1995–2013 period. They showed that there is both-way causality between CO_2_ emissions and economic growth, and between health expenditures and economic growth for the selected panel, and that there is unidirectional causal interplay from CO_2_ emissions to health expenditures, except for economies in the low-income group. In their study of Chinese provinces, Yu et al. [19] aimed to find out whether pollution increases government spending on health care. Using a panel data model for 31 provinces for the period 1997–2014, the study found that both in the long and in the short run, public health care expenditures were positively affected not only by the provincial economy, but also by the environmental quality. Lu [20] investigated the cointegration and causality relationships among greenhouse gas emissions, energy consumption and economic growth for 16 Asian countries over the period 1990–2012 and showed the existence of bidirectional Granger causality between energy consumption, GDP and greenhouse gas emissions and between GDP, greenhouse gas emissions and energy consumption in the long run. Sterpu, et al. [21] analysed the relationship between per capita greenhouse gas emissions, gross domestic product, gross inland energy consumption, and renewable energy consumption for a panel of countries in the European Union for the period 1990–2016 and derived the existence of long-run equilibrium relations among the four macroeconomic indicators, while the cubic model showed that the environmental curve was an inverted N-shape. The effect of certain environmental factors, economic factors and non-economic factors upon public and private health expenditures has been studied by Usman et al. [22] for the panel data of 13 emerging economies for the period 1994–2017. The results showed that the air-pollution indicators, CO_2_ emissions and the environment index, have a positive and significant influence on public health expenditures. By contrast, both factors negatively influence private health expenditures in emerging economies. Moreover, economic factors such as GDP growth consistently show a positive impact on both government and private health expenditures, whereas foreign direct investment exhibits a significant negative and positive impact on government and private health expenditures respectively. As for the non-economic factors, population ageing increases health expenditures, while secondary education lowers private health spending in emerging markets. In another study, Pi et al. [23] investigated the effects of air pollution, physical health, and medical insurance costs in a large sample of 23 Chinese provinces. The results show both inverted and usual U shaped curves for the effect of two pollutants (PM_10_ and NO_2_) on health expenditures. In another study related to a particular Chinese province, Liao et al. [24] estimate the health impact of PM_2.5_ in 14 cities in Gansu Province for the period 2015–2017 and showed that the presence of this particular pollutant has a negative health effect of around 6% of GDP of the provinces. In their study, To et al. [25] examined whether foreign direct investment (FDI) is causing environment degradation by testing the validity of the traditional Environmental Kuznets Curve in the context of emerging Asian markets for the period 1980–2016. The results indicated that the pollution heaven hypothesis and the Environmental Kuznets Curve are more or less valid in the region, while FDI has a strong impact on the environment. In a regional study on this topic, Li et al. [26] examined the EKC relationship between economic growth and energy consumption in the upper-middle-income regions of China with the panel data of 21 provinces for the period 2000–2017 and show that the energy consumption EKC fitting map in these regions conforms to the classical environmental Kuznets curve. In a similar study, An and Heshmati [27] analysed the interrelationship between atmospheric pollutants and healthcare spending in the monthly panel data for 16 cities and provinces in Korea between January 2010 and September 2017. The results show that the selected air pollutants, NO_2_, O_3_, and PM_10_ have a positive relationship with healthcare expenditures. Urbanization policies that aim at transforming the rural economy have also put a burden upon the health costs of countries. Diao et al. [28] capture this issue and estimate the negative health effects caused by exposure to PM_2.5_, as an indicator of urban pollution, in 338 cities of China for the year 2015. They showed that PM_2.5_ pollution remains high over the year, which causes many people to suffer from all kinds of serious health problems, especially premature death and related diseases leading to 2.73% decrease in GDP. Therefore, there is a strong positive correlation between urbanization level and health risks as well as economic loss.

### Research Gap and Objectives of the Study

The review of the extant literature does not include, in most cases, studies on the interrelationships between the three indicators of economic growth, environmental pollution and health expenditure, with the exception of Chaabouni et al. [18]. There are certain studies [4,19,23,27] which focus on the nexus between environmental pollution and different sources of health expenditures. However, no study has investigated the same through theoretical models and empirical observations for the world’s leading polluting countries. The present study tries to investigate the interrelationships between air pollution, in terms of total CO_2_ emissions, and health expenditures, as the measure of all sorts of health costs. We employ both theoretical modelling and empirical observations for the world’s top 20 polluting nations over the period 1991–2019.

## 3. Materials and Methods

### 3.1. Rationale of the Study

The short-run and long-run effects of health expenditures and environmental pollution of any sort upon the growth of per capita income through the endogenous growth model has not been taken up by any study so far, as the above review of the literature shows. Thus, the present study first analyses the effects of health expenditure and pollution of any sort upon the growth of per capita income and consumption with the help of the endogenous growth model. Then, it tests whether there are long run associations and short run interplays between health expenditure and pollution as they both explain growth of income.

### 3.2. Theoretical Model

Increases in health expenditures caused by an upsurge in income have two effects: they save lives and increase life expectancy and increase human capital formation which in turn increases labour productivity and helps further economic growth. There has been enough empirical evidence on the interrelationships between formation of human capital and growth of income [11,29,30,31]. Therefore, the linkages between income, health expenditure and pollution can be modelled in an endogenous growth framework.

There are two sectors acting independently, namely households and companies on the one hand, and the market which makes association/interactions between these two distinct sectors in a decentralized framework. We follow the intertemporal models of Ramsey [32], Cass [33] and Koopmans [34] where optimum savings (or endogenized savings rate) are derived to make capital formation for long run growth. Then we consider the implications of the benevolent social planner in our model.

On the demand side, the goal of households is the maximization of utility which is affected by their intertemporal budget constraints and by the supply side; companies maximize profits subject to the factor accumulation constraint. Then supply equals demand in the equilibrium in real terms.

Let us set the social planner’s aim as the maximization of households’ intertemporal utility subject to the aggregate budget constraint, which shows how the economy’s output is allocated to different uses. Households’ intertemporal utility function (the objective function) is:

(1) $$\begin{array}{l} u=\int_{0}^{\infty }e^{(n-\rho )t}u(c)dt \end{array}$$

where ‘*u*’ is the per capita utility, ‘*c*’ is the consumption expenditure per capita, ‘*n*’ is the growth rate of population and ‘*ρ*’ is the discount rate.

The subjective function is the state equation for capital formation which is:

(2) $$\dot{k}=f(k)-(n+\delta )k-c$$

where *k* (=d*k*/d*t*) is the rate of growth of capital per capita, *f*(*k*) is the per capita output and ‘*δ*’ is the depreciation rate.

In a two-sector closed economy without government, the aggregate outcome can be used for consumption and investment in physical capital, that is:

*Y* = *C* + *I* (3)

We know investment is the change in capital stock, d*K*/d*t*, plus amount of funds devoted to depreciation adjustment, *δK*, and the amount required to feed additional population, *nK*. Hence:

*Y* = *C* + *dk*/*dt* + *δK* + *nK* (4)

In per capita terms:

*dk*/*dt* = *f(k)* − *(n + δ)k* − *c* (5)

Considering the utility function as a constant intertemporal elasticity of substitution (*σ*), the relevant Hamiltonian for utility maximization of a social planner is:

(6) $$H\left(c,\;k\right)=\left(c^{1-\sigma }/1-\sigma \right).\;e^{\left(n-\rho \right)t}+\beta \;\left[f\left(k\right)-\left(n+\delta \right)k-c\right]$$

The answer to this problem, usually is obtained through the use of a Hamiltonian function, which gives a non-linear differential equation (taking zero growth of population, *n* = 0) that defines the optimal development of consumption expenditure in the form of:

(7) $$\begin{array}{l} \left[\left(\partial c/\partial t\right)/c\right]=1/\sigma \;\left[f_{k}-\left(\rho +\delta \right)\right]\\ or,\dot{c}/c=1/\sigma \;\left[f_{k}-\left(\rho +\delta \right)\right] \end{array}$$

where ‘*σ*’ is the intertemporal elasticity of substitution in consumption and *f_k_* is the marginal productivity of per capita capital. In the steady state, *∂c/∂t* = *∂k/∂t* = 0 which gives *k* * as the steady state capital per capita and the corresponding consumption per capita is *c* * = f(k *) − (n + δ) k * and the marginal output of per capita capital is *f_k_* * = *ρ* + *δ*.

However, the inclusion of endogenous factors like technological changes, human capital formation, public institutions leads to positive growth of per capita income and consumption and so *c*/c > 0.

Now we introduce the human capital factor into the above model. Note that an increase in health expenditure induced by an increase in income generates additional human capital, which may play an endogenous role in influencing the rate of growth of consumption and income in the long run.

Suppose the total production function is:

*Y* = *f(L, K, P)* (8)

where ‘*P*’ is the total quantity of environmental pollution. We consider here only air pollution which is represented by the total quantity of CO_2_ emissions. The other sources of pollution like water, soil, noise etc. and that of other atmospheric pollutants are not taken into account since the data on these alternative sources are not readily available in a time series format. We consider ‘*P*’ as the total quantity of CO_2_ emissions, the indicator of total environmental pollution. *Y_L_* > 0, *Y_LL_* < 0; *Y_K_* > 0, *Y_KK_* < 0. Again, in general circumstances, *Y_P_* > 0, *Y_PP_* < 0 for the developing economies which lie in the short run phase (increasing part) of EKC; but *Y_P_* < 0, *Y_PP_* < 0 for the developed economies which lie in the long run phase (decreasing part) of EKC.

Furthermore, the aggregate health expenditure (*H*) is a positive and decreasing function of income which again, ceteris paribus, is a function of pollution. In the long run, as *H* increases, the number of healthy individuals increases and human capital formation is expanded. Therefore, the general exhaustive production function is:

*Y* = *f(L, K, P, H)* (9)

Suppose a specific form of the function is considered to be:

*Y* = *AL^(1−α−β−γ)^. K^α^. P^β^. H^(1+γ)^* (10)

where 0 < *α*, *β* and *γ* < 1. The production function depicts increasing returns to scale which paves the way for positive growth of income and consumption in per capita terms in the long run.

Writing the production function in per capita terms we have:

*y* = *Y*/*L* = *AL^(1−α−β−γ)^ K^α^ P^β^ H^(1+γ)^*/*L* = *AL^(1−α−β−γ)^*/*L^(1−α)^*. *K^α^ P^β^ H^(1+γ)^* (11)

We have pollution per capita *p* = *P/L* and per capita health expenditure *h* = *H/L*. Substituting *P* and *H* in terms of their per capita levels, the per capita output is reduced to:

*y* = *AL^(1−α−β−γ)^ K^α^ (p. L)^β^. (h. L)^(1+γ)^* (12)

or,

*y* = *AL. k^α^ p.^β^. h.^(1+γ)^* (13)

In a given point of time economies maintain a fixed p (because of technology) and h (because of consumers’ and governments’ budgets) at say *p*_0_ and *h*_0_. Hence:

*y* = *AL. k^α^ p_0_.^β^. h_0_.^(1+γ)^* (14)

Now marginal productivity of per capita capital MP_k_ (f_k_) is *dy/dk* *=* *A*. *α*. *L*. *k^α^*^−1^ *p*.*^β^*. *h*. *^(1+γ)^*.

Now *df_k_/dk* > 0, not <0.

Again, *df_k_/dp* > 0 and *df_k_/dh* > 0.

Therefore:

(15) $$\dot{c}/c=1/\sigma \;\left[A.\alpha .L.\;k^{\alpha -1}p.^{\beta }.\;h.^{\left(1+\gamma \right)}-\left(\rho +\delta \right)\right]>0$$

So, with the combination of long and short run effects there can be an increase in human capital formation through an increase in health expenditures (due to an increase in income in the short run and an increase in pollution in the long run), which may lead to an increase in per capita income and consumption. If the short run benefit of the increase in health expenditures dominates the long run cost of pollution, then the growth rate of consumption per capita and growth rate of income per capita will be positive. This situation establishes the causal influence of pollution upon health expenditures. Besides, there can be a reverse causation, since an increase in health expenditure due to an increase in income may cause further pollution. The developed economies, which are in the final part of the EKC, may exhibit the first causal interplay, while the developing economies, which are in the initial part of EKC, may have second causal interplay.

It is now necessary to verify the theory with the help of empirical data on the linkage between health expenditure and pollution via income effects. We keep income as the latent variable which is embodied in heath expenditures.

### 3.3. Data and Methodology for Empirical Verifications

The study uses data from the World Bank and OECD Statistics for two main variables, CO_2_ emissions and per capita health expenditure. We consider here only air pollution which is represented by the total quantity of CO_2_ emissions, because the data on this type of pollutant is readily available in a time series format for all the countries in the list. Health expenditure is the aggregate of monetary costs borne by the citizens and governments of the selected countries for using and providing health care services. The period of study is 1991–2019. The data for CO_2_ emissions have been extrapolated for the years 2016–2019 due to non-availability of the data, and the data on per capita health expenditure (measured in current United States Dollar (USD)) for non-OECD members have been also extrapolated for the period 1991–1999 on the basis of the average growth rates of the variable for the period 2000–2019. Total CO_2_ emissions for the countries have been converted into per capita terms with the help of the population data published by the World Bank. The extrapolations of the data of the two series are done to obtain long time series data to which time series econometric tools can be applied.

The study has considered the world’s leading 20 countries in CO_2_ emissions, out of which 11 are developed and nine are developing. Since the study has 29 year/time points for both series, there may be stochastic trends in them. As a consequence, it is essential to test for stationarity (or non-unit root situation) of the two series for all the selected countries. The test for the existence of unit roots (i.e., non-stationary series) is done through the Augmented Dickey-Fuller (ADF) technique [35]. For a data set (*x_t_*, *t* = 1, 2,..., *T*), let us consider the following linear regression system for testing the unit root for two variants of the ADF(*p*) regression–viz.:

(16) $$\begin{array}{l} \;\;\;\;\;\;\;\;\;\;\;\;\;\;\;\;\;\;\;\;\;\;\;\;\;\;\;\;\;\;\;\;\;p\\ \Delta x_{t}=\delta +\beta x_{t-1}+\Sigma \gamma_{j}\Delta x_{t-j}+\varepsilon_{t}\\ \;\;\;\;\;\;\;\;\;\;\;\;\;\;\;\;\;\;\;\;\;\;\;\;\;\;\;\;\;\;\;j=1 \end{array}$$

for the without time trend case and:

(17) $$\begin{array}{l} \;\;\;\;\;\;\;\;\;\;\;\;\;\;\;\;\;\;\;\;\;\;\;\;\;\;\;\;\;\;\;\;\;p\\ \Delta x_{t}=\delta +\alpha t+\beta x_{t-1}+\Sigma \gamma_{j}\Delta x_{t-j}+\varepsilon_{t}\\ \;\;\;\;\;\;\;\;\;\;\;\;\;\;\;\;\;\;\;\;\;\;\;\;\;\;\;\;\;\;\;j=1 \end{array}$$

for the with time trend case.

If *β* = 0 (or *ρ* = 1) gets rejected by the ADF values, then we say that the series is stationary or free from the unit root problems. If this feature works for the series of per capita health expenditure (PCHEXP) and per capita pollution/per capita CO_2_ emissions (PCCO_2_), then regression of one on the other can be run without the possibilities of getting spurious outcomes. If the reverse is true, then it is essential to test whether the series are integrated of order one (I(1)) or they are stationary at their first differences. If it appears that both series are I(1), and their estimated error/noise is stationary, then we can say that both series are cointegrated and there are long run or equilibrium relationships between them. In the time series econometric literature, there are two ways of testing cointegration: the Engle-Granger method and Johansen method. The present study has examined the presence of long run relations between PCHEXP ratio and PCCO_2_ in line with Engel-Granger [36] cointegration method, and short run dynamics through the Error Correction Mechanism (ECM) and Granger Causality analysis [37].

### 3.4. Cointegration and Error Correction Mechanism

The prerequisite to investigate a cointegrating relation or equilibrium relation between the two series is that the two series should follow I(1), so that the derived error term follows I(0). Engel and Granger (EG) offered a solution to this problem by presenting the idea of cointegration. Suppose two series *y* and *x* are both I(1) and are linked by the equation as:

(18) $$y_{t}=\delta +\beta x_{t}+\varepsilon_{t}$$

and their linear combination $\varepsilon_{t}=y_{t}-\delta -\beta x_{t}$ is I(0). Then the series of *y* and *x* will be cointegrated or will have equilibrium relations between them. Thus, a genuine cointegrating relation between the two series can be derived by estimating Equation (3). After that, it is required to test the estimated error term, ${\hat{\varepsilon }}_{t}=y_{t}-\hat{\delta }-\hat{\beta }x_{t}$, to be I(0). If the series of ${\hat{\varepsilon }}_{t}$ is found to be I(0), then it can be said that the series are cointegrated in line with EG. The estimated coefficients $\hat{\delta }$ and $\hat{\beta }$ give rise to the long run equilibrium parametric values of the relation between *y* and *x*. The regression equation for the *i*th country selected for the study is:

(19) $$y_{it}=\hat{\delta }+\hat{\beta }x_{it}$$

The stationarity feature of the estimated error term, ${\hat{\varepsilon }}_{it}$, can be checked in line with the ADF test by estimating the following equation:

(20) $$\Delta {\hat{\varepsilon }}_{i,t}=\lambda {\hat{\varepsilon }}_{i,t-1}+\sum_{j=1}^{p}\phi_{i}\Delta {\hat{\varepsilon }}_{i,t-j}+u_{i,t}$$

After that, we test whether λ = 0 (null hypothesis) against λ < 0 (alternative hypothesis). If the null hypothesis (i.e., λ = 0) is rejected, then we say that both *y* and *x* are cointegrated series and an equilibrium relation between them exists.

Along with the presence of a long run relation between the two variables, there can be short run deviations, called errors, from such equilibrium relation/s. It is thus required to verify whether these errors get corrected or they converge to the equilibrium relation. If they converge to the equilibrium, then it is said that errors are corrected. The short run dynamics vis-à-vis the equilibrium relation can be modelled by the Error Correction Mechanism (ECM). The ECM can be written as follows:

(21) $$\Delta y_{it}=\delta +\eta \Delta x_{it}+\gamma {\hat{\varepsilon }}_{i,t-1}+e_{i,t}$$

Here ${\hat{\varepsilon }}_{i,t-1}\;$ stands for the error correction term and γ stands for the speed of convergence or divergence. If the estimated γ is found to be negative and significant, then the series are converging and the short run deviations are corrected, or the error is temporary. Alternatively, if the estimated γ is found to be positive and significant, then it is said that the series are diverging and are going away from the long run equilibrium relation, or the error is permanent. Equation (6) represents the short run relation between the variables and η stands for the short run regression coefficient or the rate of change in PCHEXP due to one per cent change in PCCO_2_ or the reverse.

### 3.5. Granger Causality Test

For a bivariate non stationary model with both I(1) property a Granger Causality Test is done by estimating Equations (7) and (8) in the first differenced forms of the variables including the error correction terms for *y* on *x* and *x* on *y* [37]. The model is:

(22) $$\begin{array}{l} \;\;\;\;\;\;\;\;\;\;\;\;\;\;\;\;\;\;\;T_{11}\;\;\;\;\;\;\;\;\;\;\;\;\;\;\;\;\;\;\;\;T_{12}\\ \Delta y_{t}=\mu_{yx}+\Sigma \alpha_{1j}\Delta y_{t-j}+\Sigma \beta_{1j}\Delta x_{t-j}+\eta_{yx}ecy_{t-1}+\varepsilon_{lt}\\ \;\;\;\;\;\;\;\;\;\;\;\;\;\;\;\;\;j=1\;\;\;\;\;\;\;\;\;\;\;\;\;\;\;\;\;\;j=1 \end{array}$$

(23) $$\begin{array}{l} \;\;\;\;\;\;\;\;\;\;\;\;\;\;\;\;\;\;T_{21}\;\;\;\;\;\;\;\;\;\;\;\;\;\;\;\;\;\;T_{22}\\ \Delta x_{t}=\mu_{xy}+\Sigma \alpha_{2j}\Delta y_{t-j}+\Sigma \beta_{2j}\Delta x_{t-j}+\eta_{xy}ecx_{t-1}+\varepsilon_{lt}\\ \;\;\;\;\;\;\;\;\;\;\;\;\;\;\;\;\;\;\;\;\;j=1\;\;\;\;\;\;\;\;\;\;\;\;\;\;j=1 \end{array}$$

where Δ denotes the first difference operator; *T_l m_, l, m* = 1, 2, 3 denotes the number of lagged values of Δ*y* and Δ*x* that affect the current values of these differenced variables; *μ*, *α*, *β* and *η* denote regression parameters; ε*_lt,_ l* = 1, 2 are the white noise disturbance terms. The parameters *η_yx_* and *η_xy_* in the equations are called the adjustment parameters, which are required to be negative and significant to justify the significant error correction feature. *ecy_t−1_*and *ecx_t−1_* respectively represent the error correction terms obtained from residuals of the regressions of *y* on *x* and *x* on *y*. The nature or direction of Granger Causality is determined by the values of the F statistics under the following criteria:

1. If *β_1j_* = 0, for all *j* and *η_yx_* = 0, *x* may be said not to *Granger cause y*. 2. If *α_2j_* = 0 for all *j* and *η_xy_* = 0, *y* may be said not to *Granger cause x*. 3. If (1) holds but (2) does not, *Granger causality* may be said to be *unidirectional from y to x*. 4. Conversely, if (1) does not hold but (2) does, *Granger causality* may be said to be *unidirectional from x to y*. 5. If both (1) and (2) do not hold, *Granger causality* between *x* and *y is* said to be *bi- directional or feedback,* and 6. If both (1) and (2) holds, there is no *Granger causality* between *x* and *y*.

## 4. Empirical Results

**Figure 1:**
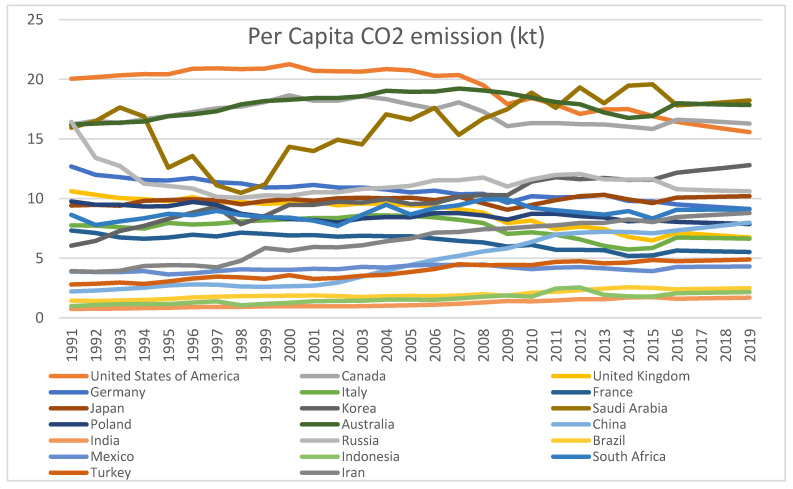
Per Capita CO_2_ emission (kt). Source: Author’s own derivations.

**Figure 2:**
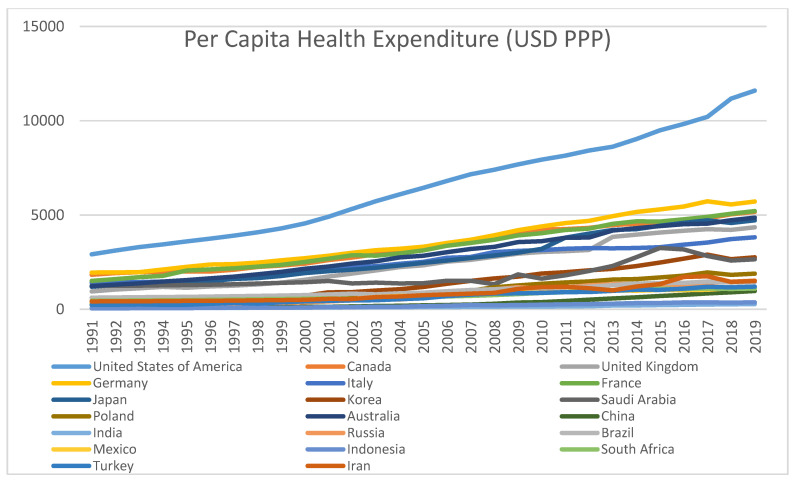
Per Capita Health Expenditure (USD PPP). Source: Author’s own derivations.

First, the scenarios of the two concerned indicators (PCCO_2_ and PHEXP) in the selected countries for the 1991–2019 period are presented to have a view on the pattern of the two series over time. Figure 1 and Figure 2 respectively present the trends of the two series for the twenty countries. It can be observed from Figure 1 that the developed countries show a falling trend of the PCCO_2_ in the new millennium after maintaining rising trends in the pre-millennium period. Some countries, like the United States of America (USA), Canada, Australia, and France, show inverted EKC shapes of their series on per capita pollution. USA, Canada, Australia and Saudi Arabia are the countries with larger quantities of PCCO_2_ in the group of 20. The so-called developing countries are in the bottom line. The difference between the developed group and the developing group in this regard is that the former maintains a falling trend of PCCO_2_ in general, while the latter group maintains an increasing trend.

From Figure 2 it can be observed that all the twenty countries show rising trends in the PCHEXP over the period of study. The developed countries in the group show higher PCHEXP compared to that of the developing group. USA is again on top of the list and India is at the bottom.

Table 1 presents descriptive statistics on the two variables with their average, standard deviation and correlation coefficient between the two for the entire period. It can be observed that the average emissions of CO_2_ per capita by the USA is the highest (near double to its closer members in the developed group) and India has the lowest of all. With respect to PCHEXP, again the USA lead the group and India is the last. USA and Canada are close to each other in health expenditure per capita whereas India and Indonesia are close to each other.

The trends of PCCO_2_ and PCHEXP (refer to Figure 1 and Figure 2) exhibit opposite directions of the movements in the two series for almost all the developed countries and positive directions for the developing group in the list. This means that there are respectively negative correlations and positive correlations for the developed and developing countries between pollution and health expenditure. The computed correlation coefficients for all the countries show the same trend. The statistical significance of these correlation coefficients has been tested by ‘t’ statistic, which shows significant results for all countries except Russia. Therefore, in general, we have the preliminary result that per capita pollution levels and health expenditures are associated/correlated for the period under study across the 20 leading polluters in the world. Hence, we need to take up the empirical analysis to find whether there are long run associations and short run dynamics between per capita emissions and health expenditure for the group of top twenty polluting countries.

The first essential task to handle time series data is to test whether the series are free from unit root problems. Table 2 presents the test results under the ADF test method, followed by Equations (1) and (2). The results show that the two series, PCCO_2_ and PHEXP, are stationary at their first differences for all countries except China, which has a second differenced stationary. The series are I(1) for all the 19 countries and I(2) for China. Therefore, we can proceed to test long run associations between pollution and health expenditure for the nineteen I(1) countries and the one I(2) country.

The long run relation or cointegration between the two series for the 19 countries and their error corrections are analysed through the EG method following Equations (3)–(6). The results of cointegration are given in Table 3.

The results show that out of 19 countries (except for China that has I(2) series for both the indicators) there are 12 countries where both per capita pollution and health expenditures have long run relations or equilibrium relations. Furthermore, out of 11 developed countries, a significant cointegration is observed in seven countries, which are USA, UK, Germany, Italy, France, South Korea and Saudi Arabia. Moreover, out of eight developing countries, except China, there are five countries (India, Brazil, Indonesia, Turkey and Iran), where significant cointegration exists. Hence, broadly speaking, pollution and health expenditure are cointegrated, which is a good outcome for the proposed study, for it satisfies our theoretical model. However, as regarding the short run dynamics, the EC results show a very limited number of countries whose associated errors due to deviations from their long run relations are corrected. This means that the deviations from the equilibrium relations are permanent for the majority of the countries where cointegration between the variables exist.

The results in the table (Table 4) show that, in the short run, there are different results overall for the developed economies vis-à-vis the developing countries. It can be observed that five countries from the developed group show causal interplays between pollution and health expenditure, which means that pollution influences health expenditure, as we claimed in our theoretical model. Canada is the only country where bidirectional causal interplays between pollution and health expenditure are observed. No other developed country has produced significant causal interplays between the two. Hence, in most cases, pollution is identified to be the influencing factor to increasing health expenditure.

Nonetheless, for the majority of the developing countries there is no such causal interplay. Only for three countries—India, South Africa and Turkey—health expenditures have an influence on the pollution level. This is what we claimed earlier in the case of the developing countries, because high health expenditure is the indicator of increase in income in the short run, which is obtained by more intense exploitation of the environment.

Therefore, the overall results for the developed countries are best suited to the long run framework and are located in the falling part of the EKC. The developing countries for which significant results are found, are located in the rising part of the EKC. The theoretical explanation confirms the empirical results.

## 5. Discussion

This study establishes theoretically, through the endogenous growth model, that both per capita health expenditures in monetary units and per capita pollution in terms of CO_2_ emissions have positive impacts upon the growth of per capita income and consumption in the general economy. This means that there have been certain interactions between per capita health expenditure and per capita pollution to justify the income growth which explain the economies’ positions along the EKC. An increase in health expenditures leads to an increase in life expectancy and thereby add to the stock of human capital, which further influences the growth of income of the countries. Furthermore, environmental pollution leads to increased production and outputs in all the sectors of the economy, which in turn bring a growth of income in the short term. This induces to spend more on pollution abatement technologies like that on conservation capital in order to obtain long run benefits in terms of sustainable development. The issue of whether these interactions between per capita health expenditures and per capita pollution work in both the long run and short run has been empirically examined in this study by considering the top 20 countries in terms of atmospheric pollution. The study concludes that both indicators follow long run or equilibrium relations, which means per capita health expenditure and per capita pollution have moved side by side over the period of study in the majority of the selected countries. Moreover, the causal interplays between the two indicators in the short run have been analysed to observe the dynamics between the two. The results show that in the majority of the developed economies pollution is identified to be the influencing factor to increasing health expenditures. On the contrary, for the majority of the developing countries there is no such causal interplay. Only for three countries— India, South Africa and Turkey—it is health expenditures which influence the pollution level. The results support what the study has claimed earlier in the case of the developing countries, because high health expenditure is the indicator of an increase in income in the short run, which is obtained by a more intense exploitation of the environment. Overall, the developed countries show results that mostly suit the long run framework and are located in the falling part of the EKC. The developing countries that show significant results are found in the rising part of the EKC. Therefore, the increasing pollution in the so- called developed countries influences a higher use of health care services as well as the provision of more such services by the governments. The results support the observations of the studies such as Narayan and Narayan [4], Chaabouni, et al. [18] regarding the cointegration and causality results at the country levels. Further, the results of the present study go some way with the results of the provincial as well as city level studies in Korea and China respectively (An and Heshmati [27]; Diao et al. [28]). The policy makers of these countries should consider reducing their pollution loads. Additionally, the results are alarming for the developing countries since they face increasing health expenditure through an increase in income which is obtained by further exploiting the environment. This supports the study by Diao et al. [28] as well for a developing country, China. Hence, on the whole, countries should focus on reducing pollution to have a sustainable development in the long run.

## 6. Conclusions and Future Research Agenda

This study examined the interlinkages between health expenditures in monetary units and environmental pollution in terms of CO_2_ emissions in a selection of the top 20 polluting countries in the world. It did so through theoretical and empirical models which demonstrate that per capita health expenditure and per capita pollution are cointegrated in the majority of the countries in the long run. However, in the short run, pollution has an influence on the health expenditure of many developed countries in the list, but it is health expenditure that influences pollution in some of the developing countries. The results justify the claim of the endogenous growth model that includes pollution and health expenditure. The results for the developed economies conform to the falling part of EKC, while those of the developing economies conform to the rising part of the EKC.

However, there is no consensus on the results for both the sets of developed and developing economies. It may be the case that fundamental differences exist between the economies in the way they grow, in their spending behaviours in health care services and the ways of using the environmental resources. This is a serious limitation of the present study.

The limitations could be resolved by considering a larger number of countries in both groups on one hand or, on the other hand, by taking a panel of the existing 20 countries. Furthermore, instead of considering only CO_2_ emissions as the indicator of pollution, the total quantity of emissions of greenhouse gases could be considered as the suitable indicator of atmospheric pollution. These two directions for further investigation can be included in our future research agenda.

## Figures and Tables

**Table 1:** Descriptive statistics. Source: Author’s own calculations.

Country	Average (PCCO_2_)	SD (PCCO_2_)	Average (PHEXP)	SD (PHEXP)	Correlation Coefficient	*t* Values (*p*)
United States of America	6516	2601.632	19.21363	1.861279	−0.89415	−10.37 (0.01)
Canada	3334	1126.657	17.06334	0.88801	−0.46232	−2.709 (0.05)
United Kingdom	2464	1161.124	8.758151	1.289833	−0.96192	−18.28 (0.01)
Germany	3625	1284.053	10.64438	0.88965	−0.9453	−15.05 (0.01)
Italy	2534	785.431	7.514357	0.856867	−0.63267	−4.245 (0.01)
France	3287	1170.555	6.38005	0.649456	−0.91989	−12.18 (0.01)
Japan	2760	1219.106	9.850593	0.298101	0.432676	2.493 (0.05)
Korea	1382	848.6826	9.933926	1.83271	0.936752	13.907 (0.01)
Saudi Arabia	1772	632.222	16.18668	2.54894	0.595657	3.853 (0.05)
Poland	989	557.6097	8.631876	0.572912	−0.72773	−5.513 (0.01)
Australia	2930	1178.464	17.84431	0.920926	0.317275	1.738 (0.06)
China	331	284.2651	4.727953	2.112316	0.931261	13.281 (0.01)
India	134	66.95031	1.190575	0.338213	0.949835	15.780 (0.01)
Russia	772	468.893	11.35594	1.238982	−0.12432	−0.651 (0.2)
Brazil	976	294.9416	1.961122	0.353308	0.94661	15.257 (0.01)
Mexico	705	275.2264	4.112457	0.219916	0.650419	4.449 (0.01)
Indonesia	186	112.3897	1.601843	0.428217	0.877965	9.529 (0.01)
South Africa	730	234.0077	8.793801	0.542375	0.410325	2.337 (0.05)
Turkey	656	336.5572	3.920033	0.734984	0.973712	22.212 (0.01)
Iran	853	419.4096	6.472487	1.658229	0.909682	11.381 (0.01)

Notes: SD means Standard Deviation, ‘*t*’ means Student *t* statistics, PCCO_2_ means per capita CO_2_ emission and PCHEXP means per capita health expenditure.

**Table 2:** Unit root test results for per capita health expenditure and per capita CO_2_ emission. Source: Author’s own calculations.

Developed Countries				Developing Countries			
Country	ADF(Prob) PCHEXP	ADF(Prob) PCCO_2_	Remarks	Country	ADF(Prob) PCHEXP	ADF(Prob) PCCO_2_	Remarks
United States of America	−3.8 (0.01)	−4.9 (0.00)	**S at 1st ∆**	China	−4.2 (0.00)	−4.7 (0.00)	**S at 2nd ∆**
Canada	−3.0 (0.05)	−4.8 (0.00)	**S at 1st ∆**	India	−3.3 (0.02)	−4.9 (0.00)	**S at 1st ∆**
United Kingdom	−4.9 (0.00)	−6.8 (0.00)	**S at 1st ∆**	Russia	−3.7 (0.00)	−6.6 (0.00)	**S at 1st ∆**
Germany	−4.7 (0.00)	−8.4 (0.00)	**S at 1st ∆**	Brazil	−4.9 (0.00)	−4.7 (0.00)	**S at 1st ∆**
Italy	−4.5 (0.00)	−4.0 (0.00)	**S at 1st ∆**	Mexico	−4.5 (0.00)	−5.4 (0.00)	**S at 1st ∆**
France	−5.5 (0.00)	−5.5 (0.00)	**S at 1st ∆**	Indonesia	−5.5 (0.00)	−5.3 (0.00)	**S at 1st ∆**
Japan	−3.8 (0.00)	−4.7 (0.00)	**S at 1st ∆**	South Africa	−3.5 (0.04)	−6.4 (0.00)	**S at 1st ∆**
Korea	−4.8 (0.00)	−4.9 (0.00)	**S at 1st ∆**	Turkey	−5.0 (0.00)	−5.7 (0.00)	**S at 1st ∆**
Saudi Arabia	−4.2 (0.00)	−3.1 (0.04)	**S at 1st ∆**	Iran	−9.1 (0.00)	−6.2 (0.00)	**S at 1st ∆**
Poland	−5.2 (0.00)	−4.6 (0.00)	**S at 1st ∆**				
Australia	−7.3 (0.00)	−3.3 (0.02)	**S at 1st ∆**				

Note: PCHEXP indicates per capita health expenditure and PCCO_2_ indicates per capita CO_2_ emissions. The results are derived under both ADF and PP tests, but the values are shown for ADF only to avoid space problems. ‘**∆**’ and ‘**S**’ stand for difference and stationary series respectively.

**Table 3:** Cointegration test results. Source: Author’s own calculations.

Developed Countries					Developing Countries				
Country	LR Reg. Coef. (Prob)	ADF of Error (Prob)	EC Terms	Remarks on Whether Cointegration Exists	Country	LR Reg. Coef. (Prob)	ADF of Error (Prob)	EC Terms	Remarks on Whether Cointegration Exists
United States of America	−1249 (0.00)	−3.3 (0.01)	0.04 (0.1) Errors not corrected	Yes	China	-	-	-	-
Canada	−586 (0.01)	−1.6 (0.46)	-	No	India	230 (0.00)	−3.4 (0.01)	−0.05 (0.4) Errors not corrected	Yes
United Kingdom	−865 (0.00)	−2.9 (0.05)	−0.1 (0.3) Errors not corrected	Yes	Russia	−47 (0.56)	0.79 (0.9)	-	No
Germany	−1364 (0.00)	−3.8 (0.00)	−0.14 (0.07) Errors are corrected	Yes	Brazil	790 (0.00)	−2.98 (0.05)	−0.1 (0.2) Errors not corrected	Yes
Italy	−579 (0.00)	−4.8 (0.00)	0.03 (0.3) Errors not corrected	Yes	Mexico	814 (0.00)	−1.2 (0.6)	-	No
France	−1657 (0.00)	−2.86 (0.06)	−0.02 (0.7) Errors not corrected	Yes	Indonesia	230 (0.00)	−3.6 (0.01)	−0.05 (0.4) Errors not corrected	Yes
Japan	1769 (0.01)	−0.88 (0.7)	-	No	South Africa	177 (0.02)	−0.85 (0.7) Errors not corrected	-	No
Korea	433 (0.00)	−2.9 (0.06)	−0.10 (0.2) Errors not corrected	Yes	Turkey	446 (0.00)	−2.99 (0.05)	−0.27 (0.02) Errors corrected	Yes
Saudi Arabia	147 (0.00)	−2.9 (0.05)	−0.11 (0.1) Errors not corrected	Yes	Iran	230 (0.00)	−2.98 (0.05)	−0.23 (0.05) Errors corrected	Yes
Poland	−708 (0.00)	−1.1 (0.7)	-	No					
Australia	406 (0.05)	0.99 (0.9)	-	No					

Notes: LR Reg. Coef. Means long run regression coefficient, ‘prob’ means probability, ADF means Augmented Dickey-Fuller, EC means error correction.

**Table 4:** Granger causality test results: Author’s own calculations.

Developed Countries H0: Pollution Doesn’t Cause Health Exp H1: Health Exp Doesn’t Cause Pollution					Developing Countries H0: Pollution Doesn’t Cause Health Exp H1: Health Exp Doesn’t Cause Pollution				
Country	F Stat	Prob.	Lag	Remarks	Country	F Stat	Prob.	Lag	Remarks
USA	0.00 2.31	0.99 0.13	2,2	No way causality	China	0.21 1.18	0.88 0.34	3,3	No way causality
Canada	5.38 3.61	0.01 0.04	2,2	Bidirectional causality	India	0.16 3.49	0.91 0.03	3,3	d(HExp) → d(CO_2_)
UK	4.41 1.01	0.04 0.32	1,1	d(HExp) → d(CO_2_)	Russia	1.71 1.00	0.20 0.38	2,2	No way causality
Germany	0.03 0.66	0.58 0.99	3,3	No way causality	Brazil	0.37 1.41	0.69 0.26	2,2	No way causality
Italy	3.32 1.67	0.05 0.21	2,2	d(CO_2_) → d(HExp)	Mexico	0.72 0.11	0.55 0.95	3,3	No way causality
France	1.90 1.74	0.16 0.19	3,3	No way causality	Indonesia	1.61 0.02	0.21 0.87	1,1	No way causality
Japan	6.65 0.73	0.00 0.49	2,2	d(CO_2_) → d(HExp)	S Africa	0.99 3.25	0.32 0.08	1,1	d(HExp) → d(CO_2_)
S Korea	2.92 0.42	0.07 0.73	3,3	d(CO_2_) → d(HExp)	Turkey	1.86 3.25	0.17 0.04	3,3	d(HExp) → d(CO_2_)
S Arabia	1.42 0.02	0.24 0.87	1,1	No way causality	Iran	1.04 0.07	0.36 0.93	2,2	No way causality
Poland	0.16 0.73	0.92 0.54	3,3	No way causality					
Australia	4.53 0.22	0.04 0.63	1,1	d(CO_2_) → d(HExp)					

Notes: H0 means Null Hypothesis, H1 means Alternative Hypothesis, HExp means health expenditure which is the PCHEXP, Prob. means probability, ‘d’ means first difference.

## Data Availability

Not applicable.
